# microRNA-146a Signaling in Alzheimer's Disease (AD) and Prion Disease (PrD)

**DOI:** 10.3389/fneur.2020.00462

**Published:** 2020-06-25

**Authors:** Walter J. Lukiw

**Affiliations:** ^1^Bollinger Professor of Alzheimer's Disease, Louisiana State University School of Medicine, New Orleans, LA, United States; ^2^LSU Neuroscience Center, Louisiana State University Health Sciences Center, New Orleans, LA, United States; ^3^Department of Ophthalmology, LSUHSC, New Orleans, LA, United States; ^4^Department of Neurology, Louisiana State University Health Sciences Center, New Orleans, LA, United States

**Keywords:** Alzheimer's disease (AD), herpes simplex virus, microRNA (miRNA), miRNA-146a-5p, NF-kB (p50/p65), prion disease (PrD), microbiome

## Abstract

The mouse- and human-brain-resident, nuclear factor kappa B (NF-κB)-regulated, micro RNA-146a-5p (miRNA-146a-5p) is an inducible, 22-nucleotide, single-stranded non-coding RNA (sncRNA) easily detected in several brain and immunological cell types, and an important epigenetic modulator of inflammatory signaling and the innate-immune response in several neurological disorders. Among all studied microRNAs, miRNA-146a-5p (typically referred to as just miRNA-146a) has been well characterized and its pathological function in progressive, age-related, and lethal human inflammatory neurodegenerative disease states is well documented. This communication will review our current understanding of miRNA-146a, its induction by the NF-kB-stimulating actions of inflammatory mediators, including the secretory products of certain microbial species such as viral vectors, and Gram-negative bacteria (such as *Bacteroides fragilis*) that are normal residents of the human gastrointestinal (GI) tract microbiome, and how miRNA-146a appears to contribute to neuro-pathological, neuro-inflammatory, and altered neuro-immunological aspects of both Alzheimer's disease (AD) and prion disease (PrD).

## Overview

**Figure 1:**
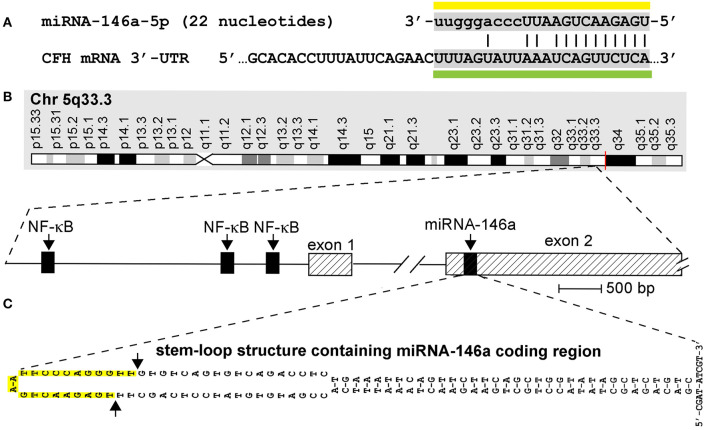
**(A)** *Homo sapiens* micro RNA-146a-5p (hsa-miRNA-146a) is a 22-nucleotide (45.5%G+C) single-stranded non-coding RNA (sncRNA) abundant in human brain and immune cells and in the central nervous system (CNS) of mice and humans (miRNA sequence overlined with yellow bar); the homology of the miRNA-146a sequence has been studied in human and several rodent and ape species, and in zebrafish [see Figure 1 in reference (13)] although the actual quenching of specific mRNA targets by miRNA-146a in most of these species still requires further analysis; miRNA-146a has several known mRNA 3′-untranslated region (3′-UTR) targets that include the 3′-UTR of complement factor H (CFH) mRNA (DNA-miRNA recognition sequence underlined with green bar; the miRNA seed sequence is indicated by capitalized ribonucleotides); CFH mRNA encodes a sialic acid-containing 383-amino-acid serum-enriched glycoprotein that plays an essential role in maintaining a homeostatic innate-immune response by modulating complement activation via acting as a soluble inhibitor of complement; CFH is one of the most abundant glycoproteins in the blood serum. CFH has a calculated molecular mass of 43 kDa; however, the apparent molecular mass of CFH in SDS-PAGE gels is approximately 55–60 kDa under reducing conditions due to extensive glycosylation (https://www.genecards.org/cgi-bin/carddisp.pl?gene=CFH; https://www.uniprot.org/uniprot/P08603); miRNA-146a ribonucleotides involved in fully complementary hydrogen bonding are indicated with an (**|**); the energy of association (E_A_; predicted energy of association for miRNA–mRNA interaction between hsa-miRNA-146a and CFH is about −24.5 kcal/mol, indicating an extremely stable miRNA–mRNA pairing (14–19); the chromosome location of the human CFH gene is chromosome 1 (chr1q31.3; GenBank accession NC_000001.11); mouse and human miRNA-146a are identical in RNA sequence (3, 15, 18). **(B)** Structural features of the miRNA-146a encoding DNA locus at chromosome 5q33.3 and details of the NF-κB-sensitive miRNA146a gene showing three upstream (5′) canonical regulatory NF-κB binding sites; as might be expected, miRNA-146a transcription is highly sensitive to induction by NF-κB (2, 3, 14, 15, 20, 21); **(C)** pre-miRNA-146a transcribed from the miRNA-146a locus has the intrinsic potential to form an extremely stable 35-base-pair stem, 60-nucleotide loop sncRNA structure (stem-loop E_A_ = −49.5 kcal/mol) with miRNA-146a encoded in the distal loop; several other secondary structures of alternate miRNA-146a-stem-loop containing configurations are possible and the 5′ and 3′ ends of pre-miRNA-146a may be significantly extended; in all other high-stability precursor miRNA-146a models, the stem-loop structure containing the mature miRNA-146a sequence is consistently located in the very distal end of the predicted loop [DNA sequence shown; upper case; highlighted in yellow and delineated by arrows on the left side of **(C)**]; RNA-polymerase II-based transcription and processing by Dicer (RNase III) of the miRNA-146a-5p precursor (pre-miRNA-146a-5p) generated by this unique stem-loop structure yields a mature miRNA-146a-5p [miRNA-146a-5p sequence over-lined with a yellow bar in **(A)**] (2, 14–16, 23). This figure is an updated and upgraded figure derived in part from a previous publication [(26); Figure 1].

One of the most important advances in modern neurobiology over the last fifteen years has been the discovery of microRNA (miRNA) in the mammalian central nervous system (CNS), and the characterization of the multiple roles of certain of these CNS-enriched miRNA signals in aging, development, longevity, and neurological health and disease (1–10). As small, soluble, amphipathic regulatory molecules that are important posttranscriptional and epigenetic regulators of messenger RNA (mRNA) abundance, speciation, and complexity, miRNAs (i) exist as 18- to 25-ribonucleotide (nt), single-stranded non-coding RNAs (sncRNAs) whose sequences are both unique and highly selected over evolution; (ii) represent the smallest information-carrying ribonucleic acids yet defined; and (iii) have been repeatedly shown to play critical and determinant roles in the onset and propagation of many human CNS disorders including progressive, incapacitating, and terminal neurological syndromes. Genome-wide analysis of all known human miRNAs, which currently number about ~2,650 unique species, has indicated that only certain miRNAs, which probably number only about ~25–30 miRNA types, (i) are abundant in the cytoplasm of human brain, retinal, and other CNS cells; (ii) are inducible by pathological factors such as pro-inflammatory cytokines and chemokines, by many different types of viral gene-encoded products and secreted bacterial exudates including the Gram-negative bacteria-derived lipopolysaccharide (LPS) and pathogenic enterotoxins (such as *fragilysin*) of many microbial species resident of the human gastrointestinal (GI) tract microbiome. Of these human brain-resident miRNA species are a selective group of inducible miRNAs that are all under transcriptional control by the pro-inflammatory transcription factor NF-kB (p50/p65). This well-characterized subset of “*rapidly activateable”* miRNAs defines a sub-family of miRNA all containing one-to-several NF-kB (p50/p65) recognition features in the immediate 5' upstream sequence of their proximal promoter regulatory regions (9–12) (Figure 1). One of these miRNAs is miRNA-146a, normally only moderately abundant in the human brain neocortex and hippocampus, but inducible to many times its basal level by pathogenic agents associated with stress, pro-inflammatory glycolipids (such as LPS), local levels of reactive oxygen species (ROS), and the abundance of Aβ42 peptides and prion amyloids whose accumulation are characteristic, respectively, of the human disorders Alzheimer's disease (AD) and prion disease (PrD) (11, 12, 14, 24–32). For example, in one study, Genechip- and microfluidic fluorescent array-based and/or LED-Northern dot blot miRNA analysis that interrogates the entire 2,650 miRNAs yet characterized in human brain tissues revealed a selective upregulation of miRNA-146a in 36/36 short post-mortem interval (PMI) human superior temporal lobe neocortical tissue specimens analyzed, compared to 30- age-, gender-, and PMI-matched controls (all PMIs < 3.1 h) (10–12, 14, 24, 25). miRNA-146a levels were found to range from 1.7- to 3.6-fold greater in AD than in age-, gender-, and PMI-matched controls in the same anatomical regions (11, 12, 14, 24, 25). Similarly, multiple independent laboratories have reported that miRNA-146a is significantly upregulated in all human prion diseases (PrD) so far examined including sporadic Creutzfeldt-Jakob disease (sCJD), Gerstmann-Straussler-Scheinker (GSS) syndrome, and fatal familial insomnia (FFI) of humans, bovine spongiform encephalopathy (BSE) of cattle, chronic wasting diseases of the family *Cervidae*, and murine and other rodent models of these prion disorders (14–20; unpublished observations). Most naturally occurring human forms of PrD represent a group of extremely rare (incidence 1–10 per 100 million) fatal, progressive, transmissible neurodegenerative encephalopathies that appear to arise following the misfolding of the cellular prion protein (PrP^C^) into an immunogenic disease-associated conformation (PrP^Sc^), or for related lethal neurodegenerations such as AD and accumulation of extremely insoluble, highly pro-inflammatory Aβ42 peptide-containing amyloids. Interestingly, as is further discussed below, there is what appears to be a strong microglial cell-mediated inflammatory and immunological component to both AD and PrD. It has been suggested that miRNA-146a may be both fundamental and integral to innate-immune or inflammatory brain cell responses in AD and PrD-mediated infections, and in other terminal neurological disorders that involve (i) the progressive and irreversible inflammatory neurodegeneration of both diseased murine and human brain tissues and cells; (ii) end-stage proteolipid accumulation; and (iii) the many altered neurochemical and neuropathological signaling processes associated with them (26–36).

## miRNA-146a Attributes in Neurological Disease

In general, the major typical mode of action of miRNAs is to interact via base-pair complementarity, base-pair recognition, and binding, with the 3′-untranslated region (3′-UTR) of their target mRNA 3′-UTRs and in doing so decrease the capability of that specific mRNA to be expressed (15, 37–40). It appears that multiple regions of single-stranded mRNAs, such as the leader sequence or the 5′-untranslated region, or introns, are capable of interacting with miRNAs, but the 3′-UTRs of mature mRNAs are preferred miRNA targets and multiple miRNAs may interact with this 3′-untranslated region, sometimes even at overlapping mRNA binding sites (15, 20, 21, 40–42). Under homeostatic neurophysiological conditions, free energies of association (**E**_**A**_) of about −20 kcal/mol or less between any miRNA and its mRNA 3'-UTR target are highly favorable, and miRNA-146a has been shown to strongly interact with multiple AD- and/or PrD-relevant mRNA targets including those mRNAs encoding the interleukin-1 receptor-associated kinase-1 (IRAK-1) and IRAK-2 (14) or complement factor H (CFH) (41, 42) (Figure 1). In addition, miRNA-146a targets both human and murine tumor necrosis factor receptor-associated factor 6 (TRAF6), a key downstream signaling protein that interacts with both IRAK1 and IRAK2 in the mediation of inflammatory signaling, although one study showed no significant changes in TRAF6 abundance in AD (14). Other notable attributes of miRNA-146a are as follows: (i) miRNA-146a is abundant in both mouse and human brain so that experimentation with miRNA-146a in both species, as well as in transgenic murine models carrying human genes, is directly comparable (42); (ii) despite about ~96 million years of evolutionary divergence between *Rodentia* and *Homo sapiens*, both mouse and human miRNA-146a have identical ribonucleotide sequences (43); (iii) miRNA-146a is particularly abundant in human brain neurons and immunological cells such as in microglial and in monocyte cells; in fact, human miRNA-146a was originally described about 14 years ago as a critical mediator of inflammatory signaling in human monocytes in the laboratory of David Baltimore (16, 17); and (iv) this miRNA has been specifically associated with an upregulation of inflammatory degenerative neuropathology in both neocortical and hippocampal regions in AD, in transgenic murine models of AD and in PrD. As such, miRNA-146a was the first “*pro-inflammatory microRNA*” characterized because its induction and upregulation are significantly linked to the onset of a dysfunctional innate-immune response and increased inflammatory signaling in multiple neurological disease states (2, 17, 18, 26).

## miRNA-146a and Inflammatory Neurodegeneration

Many if not all human diseases and microbial infections, including viral and bacterial, and prion-induced disorders appear to be initiated and/or propagated via a number of critical inflammatory components and the “*immunologically privileged*” brain and CNS are no exception (37–40). Inflammation is the body's initial response to dyshomeostasis and/or infection and is a key factor in the participation of host defenses against infectious agents, traumatic injury, and the excess of reactive oxidative stress and reactive oxygen species (ROS) that they trigger. However, disordered inflammatory signaling can also contribute to and drive the pathophysiology of chronic neurodegenerative diseases with an age-related, progressive, and inflammatory component such as those encountered in AD and PrD (29, 44). Complex intra- and extracellular interactions of the innate- and adaptive-immune systems and inflammatory mediators including microglial activation, pro-inflammatory cytokines, and chemokines coordinate critical aspects of acute and chronic inflammation. These in turn orchestrate a series of common interactive effector mechanisms of tissue inflammation that contribute interactively to angiogenesis, amyloidogenesis, remodeling of the extracellular matrix, tissue injury moderated in part by reactive oxidative species (ROS), and the recruitment of blood leukocytes that (i) in many tissues characterize the initiation of inflammation, and (ii) influence critical temporal aspects of disease initiation and progression (45–51).

AD like PrD possesses a progressive neuropathology, in part based on the aggregation of abnormal lipoprotein assemblies that result in their recognition by microglia, microglial activation, and the induction of pro-inflammatory signaling cascades that support irreversible neurodegenerative pathways. It appears that the neuroinflammation associated with AD and PrD, and other human neurodegenerative maladies may represent classical examples of an important pathological process involving cyclic, self-reinforcing, and relentless propagation of neuroglial cell activation, release of pro-inflammatory cytokines and related pathogenic factors and neuronal damage, atrophy, dysfunction, and degeneration (47–53). It has recently been suggested that the provision of highly pathogenic prokaryotic signals from the GI tract microbiome involving secreted pro-inflammatory glycolipids such as *Bacteroides fragilis* LPS and enterotoxins such as the hydrolase *fragilysin* (EC 3.4.24.74) often found in abundance in the systemic circulation and brain parenchyma of AD and/or PrD patients may well contribute to the initiation and/or propagation of these progressive and irreversible age-related neurodegenerative disease processes (20, 29, 44–46).

## miRNA-146a in AD and PrD

It may not be too surprising that the single-copy human miRNA-146a gene precursor encoded from a well-characterized chr 5q33.3 locus, with three tandem NF-kB binding sites in the immediate promoter (upstream from exon 1 of the miRNA-146a coding region), is not only extremely sensitive to NF-kB upregulation but might be an important and critical initial NF-kB-sensor for the onset and initiation of pro-inflammatory signaling in these above-described disease processes (Figure 1). The activation of NF-kB and miRNA-146a is indeed very tightly coupled and is representative of a very rapid neurogenetic signaling mechanism. Kinetically, increases in NF-kB are closely followed by increases in miRNA-146a as measured by Western and gel-shift assay, microfluidic gene array analysis, and RNA sequencing in multiple neurological cell systems (2, 14, 16, 54, 55). These experimental test systems include cytokine and Aβ42-peptide stressed human neuronal–glial (HNG) cells in primary co-culture, transgenic murine models for AD (TgAD; including the amyloid over-expressing 5xFAD TgAD model carrying 5 familial AD mutations), and in AD and PrD themselves (2, 11, 12, 14, 24–26, 31, 34). Interestingly, both the cytoplasmic and nuclear abundance of the NF-kB (p50/p65) transcription complex and miRNA-146a entity are tandemly induced by a remarkable array of multiple classes of potentially pathogenic agents such as (i) pro-inflammatory cytokines and chemokines (including IL-1β, TNFα, and other inflammatory mediators such as prostaglandins); (ii) at least three different broad classes of single- or double-stranded DNA or RNA neurotrophic human CNS- and microbiome-resident viruses ranging from herpes simplex 1 (HSV-1; Herpesviridae; dsDNA genome) to Hantavirus [HTV; Bunyaviridae; (–) ssRNA genome] to human immunodeficiency virus [HIV; Retroviridae; (+) ssRNA genome] (56); (iii) secretory products of GI tract abundant Gram-negative bacteria such as enterotoxins and LPS; (iv) neurotoxic end-stage pathogenic peptides such as the AD abundant Aβ42 peptides and related senile plaque extracts; (v) neurotrophic environmental metallotoxins that include ROS-generating aluminum and mercury sulfates; (vi) multiple biophysical agents that include traumatic shock, heat stress, and hypoxia; or (vii) any combinations of these internally, externally, or environmentally derived factors (15, 20, 31–40). These findings help define and delineate a role for miRNA-146a as an important modulator of the innate-immune response and inflammatory signaling in specific immunological and brain cell types and suggests a broad role for miRNA-146a in the brain's innate-immune signaling response, anti-neurotoxin and inflammatory response, and anti-viral immunity. Besides AD and PrD, miRNA-146a is found to be significantly increased in Aβ42 peptide- and neurotoxic metal-induced, oxidatively stressed HNG cells in primary culture and in other neurodegeneration-associated diseases such as status epilepticus (SE) (56, 57). Importantly, in diseases that have been well characterized at the molecular–genetic and epigenetic level such as AD and PrD, miRNA-146a levels are found to progressively increase with disease severity and co-localize to brain anatomical compartments enriched in inflammatory neuropathology and end-stage amyloid and proteolipid deposition (29, 31, 32, 44). This further supports the concept that an upregulated miRNA-146a and its potential for pathogenic signaling may be integral to innate immune or inflammatory brain cell responses in AD- and PrD-mediated infections and to progressive and irreversible neurodegeneration in both the murine and human brain and CNS.

## Unanswered Questions

Multiple unanswered questions remain in the study of the epigenetics and molecular neurobiology of NF-kB–miRNA-146a signaling and its pathogenic contribution to progressive and ultimately lethal age-related neurodegeneration of the mouse and human CNS. While miRNA-146a upregulation is clearly associated with the onset and/or propagation of inflammatory neurodegeneration, it is not entirely clear if this is “*the critical pathological basis*” for the support of neuroinflammation or in some cases an actual “*atypical*” neuroprotective response of target brain cells. Like NF-kB, miRNA-146a can have both pro- and anti-inflammatory effects and downstream consequences (54, 55). In HNG cells in primary culture, any externally applied stimulus, whether it be in the form of pro-inflammatory cytokines, highly neurotoxic ROS-generating aluminum or mercury sulfates, pro-inflammatory Aβ42 peptides, prion amyloids, or a viral challenge by HSV-1, or other stress factors such as hypoxia all elicit a robust pathology-inducing response. This is reflected in the rapid upregulation of NF-kB–miRNA-146a signaling over a time scale of minutes, although more chronic low-level NF-kB–miRNA-146a signaling over long time periods has also been observed in some experimental situations (2, 14, 20, 21, 41, 42). It has been further suggested that a small family of other upregulated NF-kB-sensitive miRNAs and the mRNAs of other genes whose transcription is under NF-kB-mediated transcriptional control may contribute to these pathological signaling pathways (37,52,53; unpublished observations). The fact that many of these upregulated responses involving the pathological stimulation of NF-kB (p50/p65) and miRNA-146a-5p in primary HNG brain cells, followed by highly pathogenic, chronic, and fatal consequences, are sensitive to and inhibited (i) by custom-designed metal chelators (in the case of divalent or trivalent neurotoxic metal-induced ROS and NF-kB upregulation); (ii) by anti-viral compounds such as acyclovir, zanamivir, and/or remdesivir (59; unpublished observations), (iii) by anti-NF-kB agents (22, 23, 58, 59); and/or (iv) by anti-miRNA-146a pharmacological treatment strategies that underscore the singular importance of the NF-κB (p50/p65)-miRNA-146a-mediated link in the initiation and/or propagation of pro-inflammatory signaling that supports an irreversible neurodegeneration (22, 22, 23, 59). These downstream events include the NF-kB–miRNA-146a-mediated downregulation of the IRAK-signaling system, deficiencies in CFH, ensuing dysfunction in complement signaling, and the loss of the cell's innate-immunological control (2, 13, 14, 22, 23). Indeed, well-directed ROS-quenching antioxidants and neurotoxic metal chelation, anti-viral, anti-NF-kB, anti-miRNA-146a (antagomir) pharmacotherapeutic approaches, or any combinations of these may be of use in the clinical management of AD, PrD, and other related progressive and terminal neurodegenerative disease processes with an inflammatory component (15, 22, 22, 23, 54, 55, 57–62). Very recently, intranasal administration of a miRNA-146a antagomir was shown to diminish pathological processes and improve cognitive impairment in an APP/PS1 AD transgenic mouse model (13).

## Concluding Remarks

Recent and emerging data collectively support the observation that an inducible NF-kB (p50/p65)-miRNA-146a signaling system is upregulated in neurological disorders associated with progressive, age-related inflammatory neurodegeneration including AD. This pro-inflammatory transcription factor–miRNA pairing also displays increased activity in a wide range of murine and human viral and PrD-based infections. These same data suggest that a significant pathological upregulation of NF-kB (p50/p65)–miRNA-146a signaling coupled to downregulation of pathogenic mRNA targets such as IRAK and CFH may directly contribute to altered innate-immune or inflammatory responses in both viral- and prion-mediated infections, features that also accompany progressive, irreversible inflammatory neurodegeneration in a surprisingly wide range of human brain diseases including AD and PrD and in murine transgenic models for these diseases. Currently, there is (i) considerable research effort involving miRNA-based pre-symptomatic predictive and/or diagnostic biomarkers in the cerebrospinal fluid (CSF) and blood serum of the systemic circulation; and (ii) related therapeutic opportunities that address a wide range of these insidious, progressive, and lethal neurological disorders in which signaling along the NF-kB–miRNA-146a axis appears to be playing a critical role in brain cell fate (8, 9, 24, 56). Although much of the data obtained to date are relatively preliminary and need to be further confirmed in suitable brain cell-culture models, transgenic animal model studies, and controlled clinical trials, they draw considerable and thought-provoking attention to a rapidly evolving field of neurobiology and neurogenetics that should acquire increasing relevance in the future as to the nature of the pathogenic signaling mechanisms involved in these insidious and lethal neuropathological states.

## Data Availability Statement

The raw data supporting the conclusions of this article will be made available by the authors, without undue reservation, to any qualified researcher, and are also in the process of being compiled and organized into manuscript form and will available in an upcoming publication “manuscript in preparation” (Autumn, 2020).

## Ethics Statement

All acquisition, handling, experimental and analytical procedures involving postmortem human brain tissues were carried out in an ethical manner in strict accordance with the ethics review board policies at brain and tissue donor institutions and at the Louisiana State University (LSU) Health Sciences Center. Informed consent from next of kin was obtained at brain and tissue donor institutions for all tissue samples prior to autopsy and donation; coded postmortem brain tissue samples (containing no personal identifying information of the donors) were obtained from brain tissue banks listed in the Acknowledgments section. The ethical use of postmortem human brain tissues and their analyses were also carried out in strict accordance with the Institutional Biosafety Committee and the Institutional Review Board Committee (IBC/IRBC) ethical guidelines IBC#18059 and IRBC#6774 at the Louisiana State University Health Sciences Center (LSUHSC), New Orleans, LA 70112, United States.

## Author Contributions

WL compiled and distilled the results from all laboratory experiments and those of many collaborators both domestic and international, and performed literature searches of recent peer-reviewed publications in this microRNA area of neurobiological research and wrote the article.

## Conflict of Interest

The author declares that the research was conducted in the absence of any commercial or financial relationships that could be construed as a potential conflict of interest.
